# Sex Differences in Wheezing During the First Three Years of Life After Delivery via Caesarean Section

**DOI:** 10.3390/children12081071

**Published:** 2025-08-14

**Authors:** Evangelia Papathoma, Theodore Dassios, Maria Triga, Sotirios Fouzas, Gabriel Dimitriou

**Affiliations:** Department of Paediatrics, University of Patras, GR26504 Patras, Greece

**Keywords:** caesarean section, children, wheezing

## Abstract

**Highlights:**

**What are the main findings?**

- Girls delivered by caesarean section had a higher likelihood of developing preschool wheezing compared to those born vaginally.
- Wheezing within the first three years of life was more common in boys born by caesarean section compared to girls.

**What is the implication of the main finding?**

- Our findings could assist in counselling of families in relation to the likelihood of future wheezing according to method of delivery.
- Our results highlight altered microbial colonisation as a potential mechanism for the development of wheezing in early childhood following birth with caesarean section.

**Abstract:**

Background: Emerging evidence suggests that delivery by caesarean section predisposes to wheezing in early childhood, but the effect may differ between boys and girls. Such sex-specific differences remain insufficiently explored to date, particularly considering the wide range of perinatal and antenatal confounding factors. In this study, we aimed to investigate sex-specific differences in the association between delivery by caesarean section and preschool wheezing. Methods: This is a secondary analysis of a population of 470 children (53% boys), born and cared for between August 2009 and March 2011 at the maternity services of the University Hospital of Patras, Greece. Participants were followed up regularly until the age of 36 months. Wheezing was defined as at least one episode of doctor-diagnosed wheezing per year during the surveillance period of three years. Multivariable regression models were used to explore possible associations and adjust for confounders. Results: The rate of caesarean section was 51% (*N* = 240). Wheezing was reported in 144 (31%) of the children. Following delivery by caesarean section, 52 of 137 (38%) of the boys and 33 of 103 (32%) of the girls developed wheezing. In the whole cohort, development of wheezing was significantly associated with male sex [odds ratio: 1.83 (95% CI: 1.22–2.75), adjusted *p* = 0.004], but not with caesarean section or gestational age. In girls, the development of wheezing was significantly associated with caesarean section [odds ratio: 2.48 (95% CI: 1.28–4.83), adjusted *p* = 0.007]. In boys, the development of wheezing was not significantly associated with delivery by caesarean section. Conclusions: Girls born by caesarean section developed wheezing more frequently than their vaginally born counterparts during the first three years of life. Although male sex proved an overall predisposing factor to preschool wheezing, boys born by caesarean section were not diagnosed with wheezing more frequently than those delivered vaginally.

## 1. Introduction

Delivery by caesarean section (CS), either elective (planned, non-urgent) or emergency (unplanned, urgent), has been linked to respiratory symptoms in later childhood. This is thought to occur because, as opposed to the natural process of birth through the birth canal (vaginal delivery), CS bypasses exposure to maternal vaginal flora, potentially affecting microbial colonisation and leading to altered immune regulation [1,2]. The increased risk for childhood asthma in children delivered by CS has been well-established in epidemiological studies and independent meta-analyses, which have reported that children delivered by CS have a 20% increase in the risk of subsequently developing asthma [3,4]. On the other hand, the incidence of asthma in childhood is higher in boys during pre-pubertal childhood, while girls surpass boys in asthma prevalence after puberty [5]. Few studies have investigated the impact of biological sex on the risk of wheezing following delivery via CS. These studies consistently reported a higher risk for wheezing and asthma in girls delivered by CS compared to boys [6,7]. These sex-specific differences are not entirely understood, as we are not aware of any proven mechanism that could account for a preferential transfer of intestinal microbes in girls, nor that girls differ considerably in their immunological profile compared to boys.

The studies reporting a higher incidence of asthma in girls following CS delivery are mostly large epidemiological reports based on heterogeneous populations [6,7]; their major limitation is the inability to account for a multitude of prenatal (e.g., pregnancy complications), perinatal (e.g., respiratory morbidity), and antenatal (e.g., breastfeeding characteristics) confounding factors because such information is difficult to be reliably collected at the population level. For instance, the CS rate in these studies ranged between 13 and 15% [6,7], which is considerably lower than the rate reported in most southern European countries [8]. Since young boys have a higher baseline risk of asthma compared to girls, it is possible that the actual effect of CS on wheezing could appear weaker in a setting of an already decreased CS rate.

In this study, we aimed to examine sex-specific differences in the association between delivery by caesarean section and preschool wheezing, using a consistent, single-centre cohort characterised by a high caesarean section rate, uniform perinatal care, and adjustment for multiple antenatal and perinatal confounders

## 2. Methods

### 2.1. Type, Duration, and Location of the Study

This was a secondary analysis of a prospective observational cohort study of children born after 34 completed weeks of gestation and cared for at the maternity facilities of the University Hospital of Patras, Greece, between August 2009 and March 2011. The original study was designed to explore the association of CS with the development of food allergy and atopic dermatitis in the first three years of life [9].

### 2.2. Study Population and Design

The infants were postnatally recruited in the maternity ward during daytime working hours. Exclusion criteria included chromosomal or major congenital anomalies and infants already admitted to the Neonatal Intensive Care Unit. All children were followed up by structured telephone interviews, twice per year (every six months) up to the age of three years. Participants who did not attend their first or last interview or missed two consecutive interviews were excluded.

### 2.3. Data Collection

The following information was collected. *Prenatal*: maternal age and history of maternal or paternal atopy. *Pregnancy*: gestational diabetes mellitus [10], hypertensive disease of pregnancy [11], and exposure to tobacco smoking in pregnancy. *Delivery*: biological sex at birth, gestational age, birth weight [12], mode of delivery, meconium-stained amniotic fluid, rupture of membranes exceeding 18 h [13], and Apgar score at 10 min. Caesarean section was classified as elective if it was planned and performed before the onset of labour, and emergency if not. *Neonatal outcomes*: Subsequent admission to neonatal care, type (mixed or exclusive), and duration of breastfeeding. *Follow-up outcomes* were collected after hospital discharge by structured telephone interviews at 1, 6, 12, 18, 24, 30, and 36 months of age. They included wheezing diagnosed by a clinician (doctor-diagnosed wheezing) [14], lower respiratory tract infection confirmed by a clinician [15], and use of beta-2 receptor agonists (inhalers) or oral corticosteroids [16]. The respective items were chosen by the investigators based on the current literature [10,11,12,13,14,15,16]. The telephone interviews were performed by the same investigator (EP).

### 2.4. Ethical Considerations

The study was approved by the Research Ethics Committee of the University Hospital of Patras, Greece (decision no. 476/05.12.2008). Written informed parental consent was obtained before enrolment.

### 2.5. Statistical Analysis

This was a secondary analysis of a previous cohort study, and as such, an a priori sample size estimation was not undertaken. The original sample size estimation has been reported in detail in the original study [9].

Continuous data were found to be non-normally distributed (Kolmogorov–Smirnov test) and were, therefore, presented as median and interquartile range (IQR). The primary outcome was defined as at least one episode of doctor-diagnosed wheezing per year during the surveillance period of 3 years. Comparison was made between the incidences of developing wheezing in infants delivered by CS versus those delivered vaginally using the chi-square test. Continuous variables were compared in infants who developed wheezing versus those who did not, using the Mann–Whitney U non-parametric test. Binary variables were compared between infants who developed wheezing versus those who did not, using chi-square testing. The parameters that were significantly different (*p* < 0.1) were inserted into a multivariable binary regression model with wheezing development as the outcome variable. The multivariable regression was also performed separately in the two groups of boys and girls. Multi-collinearity among the independent variables in the regression analysis was assessed by examination of a correlation matrix for the independent variables. The level of statistical significance was set at *p* = 0.05. Statistical analysis was performed using Statistical Package for Social Sciences software, version 27.0 (SPSS Inc., Chicago, IL, USA).

## 3. Results

**Figure 1:**
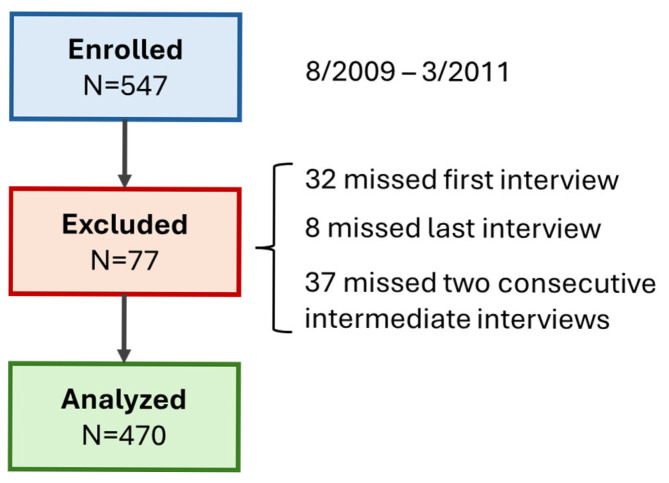
Study flow.

Of the 547 enrolled children, 470 were included in the analysis. The causes of exclusion are presented in the flow diagram of Figure 1. The characteristics of the included children (53% male) are presented in Table 1. They had a median (IQR) gestational age of 38.5 (37.4–39.6) weeks and a birth weight of 3.20 (2.86–3.50) kg. The rate of delivery by CS was 51%, and the rate by elective CS was 44%. Wheezing was reported in 144 children up to the age of three years (31%). The incidence of wheezing was higher in infants delivered by CS (35%) compared to infants delivered vaginally (26%, *p* = 0.027) and in male (64%) compared to female infants (36%, *p* = 0.003). From the children delivered with CS, 52 of 137 (38%) of the boys developed wheezing, and 33 of 103 (32%) of the girls.

The median (IQR) gestational age was lower in children who developed wheezing [38.0 (37.1–39.2) weeks] compared to the ones who did not [38.6 (37.5–39.6) weeks, *p* = 0.028]. The incidence of wheezing was not associated with a history of maternal or paternal atopy, gestational diabetes, hypertensive disease of pregnancy, maternal smoking, meconium-stained amniotic fluid, prolonged rupture of membranes, or breastfeeding. The maternal age, birth weight, birth weight z-score, and Apgar score were not different in children who developed wheezing compared to those who did not. The incidence of food allergy was not different in children with wheezing compared to children without wheezing.

Following multivariable regression analysis in both males and females, the development of wheezing was significantly associated with the male sex [odds ratio: 1.83 (95% Confidence Intervals: 1.22–2.75), adjusted *p* = 0.004], but not with CS (adjusted *p* = 0.062) and gestational age at birth (adjusted *p* = 0.27).

In females, the development of wheezing was significantly associated with CS [odds ratio: 2.48 (95% CI: 1.28–4.83), adjusted *p* = 0.007], but not with gestational age (adjusted *p* = 0.81). In males, the development of wheezing was not significantly associated with CS (adjusted *p* = 0.81) or gestational age at birth (adjusted *p* = 0.15).

## 4. Discussion

In this prospective observational study, it was confirmed that wheezing within the first three years of life is more common in boys, while birth by CS appears to have no significant association with airway hyperreactivity. However, subsequent sex-specific analysis revealed that girls delivered by CS have a higher likelihood of developing preschool wheezing compared to those born vaginally—an association that remained significant after adjusting for multiple perinatal and antenatal factors, such as gestational age, neonatal morbidity, admission to neonatal care, breastfeeding, and early-life respiratory infections. Conversely, the association between CS and wheezing was not significant among the boys of our cohort, thus underscoring the existence of sex-specific disparities between delivery mode and the development of airway hyperreactivity in early childhood.

These findings are in line with previously published reports. A cohort study of 8953 children aged 3–10 years from the United States found that delivery by CS was associated with a subsequent diagnosis of asthma in girls (odds ratio of 1.53) but not in boys [6]. Another study of 11,147 Danish children followed up to 15–18 years described a stronger association between CS and asthma among girls, albeit with marginal statistical significance [17]. In a larger cohort of 37,171 children from Norway with a follow-up of 36 months (i.e., similar to our study), the investigators reported that the risk ratio for asthma was 1.32 for girls delivered by CS as compared to a risk ratio of 1.09 for boys [7]. Most recently, in a population of 17,075 children from the United States followed up to 6 years, stronger estimated associations between CS and asthma were reported for girls compared to boys [18]. These investigators also performed a meta-analysis of five studies (approximately 60,000 children), which showed a 26% increase in the odds of asthma among girls born by CS compared to an 8% increase among boys, thus providing solid evidence on the existence of sex-specific disparities between delivery mode and wheezing in early childhood [18]. The larger sample of this meta-analysis allowed for further subgroup analyses and conclusions, such as that the observed differences were not due to the higher baseline asthma risk [18]. The present study contributes to the existing knowledge by validating the above findings within a consistent, single-centre cohort characterised by a high percentage of CS (51%, with 86% being elective), and by adjusting for multiple perinatal and antenatal confounders, not available in the reports mentioned above.

Sex differences in the epidemiology of asthma have been consistently documented in the literature. Wheezing typically shows a higher prevalence in boys during childhood, which reverses in adolescence, with the symptoms becoming more frequent in girls [19]. Similarly, boys more frequently develop early-onset wheezing, whereas girls tend to exhibit new-onset wheezing later in life, usually after puberty [20]. A study from Patras, Greece—i.e., from the same region as our research—not only confirmed these sex-related differences but also demonstrated a steady increase in the male-to-female ratio among prepubertal children with asthma between 1978 and 2008 [21]. The mechanisms that determine these patterns are not entirely understood; they may include hormonal factors [19], differences in the contribution of parental atopy according to child’s sex [20], and other biological or genetical influences, such as sex-specific expression of genetic polymorphisms, differences in immunity and lung growth in relation to sex and age, as well as in environmental exposures and behavioural/social differences [19,20,21,22].

The exact pathophysiological mechanisms underlying the sex differences in wheezing and asthma following birth by CS remain more elusive. According to the hygiene hypothesis, children who are not delivered vaginally may have altered microbial colonisation after birth, which could affect the development of the immune system and asthma susceptibility in the long term [1,2]. Our group has previously reported that CS delivery may predispose to allergic disorders in infancy and early childhood, presumably due to alterations in the establishment of normal gut microbiota [9]. The composition of the gut microbiome has been shown to differ between allergic and non-allergic children, and these differences may become apparent before the development of allergic disorders, including airway hyperreactivity and wheezing [23,24]. Recent evidence also suggests that sex differences in the establishment of gut microbiota after birth are evident and can be further influenced by factors such as mode of delivery (vaginal vs. caesarean), feeding type (breast milk vs. formula), and parity [25,26]. Sex-specific gene expression may influence gut maturation and development differently in boys and girls, while hormonal factors (e.g., oestrogens) may have a different impact on the intestinal function of female and male infants after birth [25,26]. Therefore, although the precise pathophysiological mechanisms underlying sex differences in wheezing and asthma following CS remain incompletely understood, they are most likely driven by a complex interplay of factors that lead to an altered postnatal microbial environment, thus influencing immune system development and asthma susceptibility later in life. These factors seem to have a more pronounced impact on girls born by CS, potentially making them more vulnerable to developing wheezing and asthma during infancy and early childhood.

Other potential explanations, such as that CS would place newborn infants at risk for neonatal respiratory morbidity, thus increasing the risk for subsequent development of wheezing disorders [27], were not confirmed in the present study. One may also suggest that sex-specific differences do not reflect real biological phenomena but are the partial product of methodological flaws of the analysis. If, for example, biases by unmeasured confounders affect one sex more strongly than the other, it is possible that the actual effect cannot be accurately measured or compared. One important limitation of previous reports [6,7,17,18] is the inability to account for the exact respiratory morbidity after birth or essential antenatal risk-modifying factors like breastfeeding, as this information is not always reliably collected at a population level. In the present study, using a homogeneous, single-centre population and performing a regular 3-year follow-up, it was possible to control for multiple perinatal and antenatal factors, such as gestational age, intrauterine growth and birth weight, neonatal morbidity (respiratory distress, infections, etc.), admission to neonatal care, breastfeeding, and early-life respiratory infections.

Of note, the sex differences in developing wheezing after CS were confirmed in a population with very few mothers with prolonged rupture of membranes and a considerable proportion of elective CS, unlike previous studies [6,7,17,18]. The persistence of these findings in our population implies that colonisation is indeed an essential step in this process because if CS can cause wheezing via bypassing gut colonisation, one would expect a strong association between CS and wheezing only in CS that were not complicated by prolonged rupture of membranes.

The present study has several strengths. First, it was possible to conduct detailed follow-up at several time points and collect longitudinal information on the primary outcome of interest (i.e., wheezing). Second, unlike previous reports [6,7,17,18], the primary outcome was defined as “at least one episode of doctor-diagnosed wheezing”, which is in line with the current guidelines regarding the diagnosis and management of preschool wheeze [22]. Third, as already mentioned, we accounted for multiple perinatal and antenatal confounders. Finally, although a high percentage of predominantly elective CS is not an ideal healthcare system indicator, this population was utilised to explore interactions between the mode of delivery and the development of complications in early childhood more accurately [28].

Inevitably, the study also has limitations. Its single-centre nature resulted in a cohort that was smaller compared to large population cohorts. We were, however, able to consistently collect multiple confounders and adjust accordingly in our analysis, and we could guarantee uniformity of care and minimise the unpredictable and potentially blunting effect of various confounders of inconsistent strength on the main outcome of interest.

In conclusion, we have reported that delivery by caesarean section was associated with a later development of wheezing during the first three years of life in girls. Boys delivered by section were not diagnosed with wheezing more frequently than boys delivered vaginally.

## Figures and Tables

**Table 1:** Characteristics of the study population, *N* = 470.

Prenatal	Maternal age (years)	31 (27–34)
	Maternal atopy	66 (14)
	Paternal atopy	55 (12)
Pregnancy	Gestational diabetes mellitus	57 (12)
	Hypertensive disease of pregnancy	12 (3)
	Maternal smoking	143 (30)
Delivery	Male sex	250 (53)
	Gestational age (weeks)	38.5 (37.4–39.6)
	Birth weight (kg)	3.20 (2.86–3.50)
	Birth weight z-score	−0.01 (−0.62–0.64)
	Caesarean section	240 (51)
	Elective caesarean section	205 (44)
	Meconium-stained amniotic fluid	34 (7)
	Prolonged rupture of membranes	18 (4)
	Apgar score at 10 min	9 (8–10)
Neonatal	Admission to neonatal care	28 (6)
	Breastfeeding	158 (34)
Follow up	Wheezing	144 (31)
	Lower respiratory tract infection	98 (21)
	Beta 2 agonists	259 (55)

Data represent medians with interquartile range (in parentheses) of number of cases (%), as appropriate.

## Data Availability

The raw data supporting the conclusions of this article will be made available by the authors on request.
